# Suicide methods and severe mental illness: A systematic review and meta‐analysis

**DOI:** 10.1111/acps.13759

**Published:** 2024-10-01

**Authors:** M. Trott, S. Suetani, U. Arnautovska, S. Kisely, M. Kar Ray, T. Theodoros, V. Le, S. Leske, M. Lu, R. Soole, N. Warren, D. Siskind

**Affiliations:** ^1^ UQ Medical School, Faculty of Medicine The University of Queensland Brisbane Queensland Australia; ^2^ Physical and Mental Health Research Stream Queensland Centre for Mental Health Research Brisbane Queensland Australia; ^3^ Metro South Addiction and Mental Health Service Metro South Health Brisbane Queensland Australia; ^4^ Institute for Urban Indigenous Health Brisbane Queensland Australia; ^5^ Queensland Brain Institute The University of Queensland St Lucia Queensland Australia; ^6^ School of Medicine and Dentistry Griffith University Nathan Queensland Australia; ^7^ Department of Psychiatry, Community Health and Epidemiology Dalhousie University Truro Nova Scotia Canada; ^8^ School of Health and Medical Sciences University of Southern Queensland Toowoomba Queensland Australia; ^9^ UQ Poche Centre for Indigenous Health The University of Queensland Brisbane Queensland Australia; ^10^ Australian Institute for Suicide Research and Prevention, World Health Organization Collaborating Centre for Research and Training in Suicide Prevention, School of Applied Psychology Griffith University Brisbane Queensland Australia

**Keywords:** bipolar, depression, mental health, schizophrenia, suicide

## Abstract

**Introduction:**

People with severe mental illness (SMI) have a higher risk of suicide compared with the general population. However, variations in suicide methods between people with different SMIs have not been examined. The aim of this pre‐registered (PROSPERO CRD42022351748) systematic review was to pool the odds of people with SMI who die by suicide versus those with no SMI, stratified by suicide method.

**Methods:**

Searches were conducted on December 11, 2023 across PubMed, PsycInfo, CINAHL, and Embase. Eligible studies were those that reported suicide deaths stratified by SMI and suicide methods. Studies were pooled in a random‐effects meta‐analysis, and risk of bias was measured by the Joanna Briggs Institute checklist.

**Results:**

After screening, 12 studies were eligible (*n* = 380,523). Compared with those with no SMI, people with schizophrenia had 3.38× higher odds of jumping from heights (95% CI: 2.08–5.50), 1.93× higher odds of drowning (95% CI: 1.50–2.48). People with bipolar disorder also had 3.2× higher odds of jumping from heights (95% CI: 2.70–3.78). Finally, people with major depression had 3.11× higher odds of drug overdose (95% CI: 1.53–6.31), 2.11× higher odds of jumping from heights (95% CI: 1.93–2.31), and 2.33× lower odds of dying by firearms (OR = 0.43, 95% CI: 0.33–0.56). No studies were classified as high risk of bias, and no outcomes had high levels of imprecision or indirectness.

**Conclusion:**

These findings could inform lethal means counselling practices in this population. Additionally individual, clinical, community and public health interventions for people with SMI should prioritise, where feasible, means restriction including access to heights or drugs to overdose.

**Summations**

- People with severe mental illness are more likely to die by suicide by jumping from heights than people with no diagnosed severe mental illness. In sub‐group analyses, this was also respectively significant for schizophrenia, bipolar disorder and major depression.
- People schizophrenia also higher odds of drowning as a method of suicide, and people with major depression had higher odds of drug overdoses and dying by suicide via firearms.
- The prevalence of suicide methods differ by type of severe mental illness, with suggestive time‐trends indicating that the prevalence of drug overdoses in people with schizophrenia decreases per year of publication.

**Limitations**

- Because of the low number of studies, we were unable to control for important factors, such as age, gender and cultural variables.
- Some outcomes had high heterogeneity, which could not be fully explained.

## 1 INTRODUCTION

Suicide is associated with a significant burden of disease globally. It was the leading cause of age standardised years of life lost (YLLs) in high‐income countries in the Asia Pacific and was in the top 10 leading causes of age standardised YLLs in eastern, central and western Europe, central Asia, Australasia, southern Latin America and North America in the 2016 global burden of disease (GBD) study.^1^ Severe mental illnesses (SMIs), including major depressive disorder, bipolar disorder and/or schizophrenia, are associated with an increased risk of suicide compared with the general population.^2^, ^3^ For example, a large umbrella review noted that having a psychotic disorder increased suicide mortality risk by over 13 times.^4^ Furthermore, the risk of suicide was 20 times higher in people with schizophrenia and 19 times higher in people with bipolar disorder compared with those without SMI in a 2022 systematic review and meta‐regression.^5^ The method of suicide in people with SMI has also been explored in reviews. For example, Fu et al.^6^ located 10 studies presenting data on suicide methods for people with SMI in their systematic review and meta‐analysis, and reported that the most common suicide methods amongst people with SMI were poisoning, hanging, ‘other methods’ and jumping. Of note, there was no stratification of risk by individual SMIs in this review. Importantly, SMIs are one of many contributing factors to suicide risk. Important factors such as age and gender have been well established, however other cultural and social factors (such as religiousness and social support) also contribute,^7^ indicating that the risk of suicide is part of a complex system.

One limitation of the current suicide‐SMI related evidence base is that they do not directly compare rates of suicide to those without SMI, so the pooled estimates are currently unknown. Several primary studies, however, have compared methods of suicide in people with SMI versus people with no diagnosed SMI. Recently, for example, Currie et al.^8^ found that in the US, individuals with previous psychiatric diagnoses represented nearly 40% of those who died by suicide and were ‘over‐represented’ in those who died by poisoning compared with those who used firearms. There is also evidence suggesting that people with psychiatric diagnoses are more likely to use methods with higher case‐fatality rates, such as hanging and jumping.^9^, ^10^, ^11^ Knowing which suicide methods are used by people with different SMIs is especially important given that different suicide methods have varying case‐fatality rates,^12^ and will evidence supporting means restriction across sub‐types of SMI is highly warranted.^13^ Such an approach also concurs with recommendations in the literature,^14^ which suggests that separate suicide risk prediction tools are developed for different SMI populations, such as those with mood or psychotic disorders. To our knowledge, however, there has been no previous study that statistically pooled methods of suicide across different types of SMIs compared with populations with no diagnosed SMI, therefore precluding the high‐quality evidence needed to support the development of the aforementioned SMI sub‐type‐specific suicide risk prediction tools.

The aim of this systematic review, therefore, is to synthesise the current literature base examining suicide methods, stratified by sub‐types of SMI and comparing these to people with no diagnosed SMI.

## 2 MATERIAL AND METHODS

### 2.1 Registration

We reported this study per the Preferred Reporting of Items for Systematic Reviews and Meta‐Analyses (PRISMA) 2020 guidelines^15^ and followed a pre‐published protocol (PROSPERO CRD42022351748).

### 2.2 Search strategy

Searches were conducted on the 11th of December 2023 in CINAHL, Embase, PubMed and PsycInfo. The full search strategy can be found in Table S1. Titles and abstracts were then imported into Covidence Systematic Review software, which automatically excluded duplicates.^16^ The titles and abstracts of remaining studies were then screened independently by several members of the review team (VL, SS, NW, KV SL and MT), with senior researchers (DS and SK) resolving any disputes. Reference lists of relevant articles were also searched. Following title and abstract screening, full texts of articles were retrieved and screened independently (SS, SL, MT, VL, NW and KV). For all stages of screening, the following inclusion criteria were used:

- Population: people with SMI including major depressive disorder, bipolar disorder, or schizophrenia. Studies that included specific populations (such as people incarcerated or patients in inpatient care) were excluded.
- Exposure: suicide deaths, stratified by method.
- Comparator: People with no evidence of SMI diagnosis gathered from the general population.
- Outcome: People with SMI versus people with no diagnosed SMI diagnosis who died by one specific mode of suicide versus other methods.
- Study design: all types of observational studies. Reviews were included in the title and abstract phase of screening so that reference lists can be examined for identification of other, potentially relevant studies.

### 2.3 Statistical analyses

For all analyses, we conducted a random effects meta‐analysis using the DerSimonian method,^17^ with studies weighted on their inverse variance, using R.^18^, ^19^ Only outcomes that had *k* = >2 studies were meta‐analysed. Bonferroni adjustment was used to control for multiple comparisons, with significance set at *p* = <0.002. Publication bias was assessed using the Egger's intercept with outcomes with *k* = >10 studies and the visual inspection of funnel plots (scatter plot with each point representing the respective effect size plotted against the standard error, with asymmetrical plots being indicative of publication bias) for all remaining outcomes with *k* = <10 studies.^20^ Heterogeneity was assessed using the *I* ^2^ statistic (with >50%; 51%–75%; and >75% being respectively deemed as low, moderate or high heterogeneity).^21^ Prediction intervals (PIs) were also calculated. Furthermore, a random effects meta‐analysis of prevalence proportions of suicide method, stratified by type of SMI, was conducted, with results normalised to reflect relative percentages per SMI group.

### 2.4 Data extraction and preparation

All data were extracted by two review authors (MT and SS). To determine whether suicide methods were different for people with SMI versus no SMI diagnosis, all raw data from eligible studies were converted into odds ratios (ORs), with subsequent analysis conducted. Analyses were also conducted across sub‐types of SMI (i.e., major depressive disorder, bipolar disorder and schizophrenia). We also conducted an additional analysis dichotomizing suicide methods into violent (hanging, shooting, jumping from heights, moving train, cutting, drowning and other methods) and non‐violent (drug overdose, gas poisoning or other poisoning), as per the classifications used in previous literature.^22^ If data were missing, corresponding authors were contacted via email, with a 2nd attempt made if no response was received within 2 weeks.

### 2.5 Sensitivity analyses

Several sensitivity analyses were conducted. Firstly, general sensitivity analysis was carried out using the one‐study‐removed method. Secondly, because of the differences in availability of firearms in the US compared to the rest of the world, sub‐group analyses were conducted for all outcomes that involved firearms. Third, due to geographical trends in suicide methods, sub‐group analyses were conducted on geographic region. Lastly, exploratory meta‐regressions were conducted to determine if cohort mean age, gender, or year of publication moderated significant effect sizes.

### 2.6 Study quality and credibility of evidence

Risk of bias was assessed using the Joanna Briggs Institute (JBI) checklist for prevalence studies^23^ independently by two members of the review team (SS and MT). The credibility of all results was classified according to the GRADE criteria, based on guidelines proposed by Schünemann et al.^24^ For this review, observational studies started at ‘low’ risk of bias and were upgraded due to large effect sizes (defined in this study as OR >2.0 or <0.5 and PI excluding the null). Studies were downgraded due to high risk of bias (the majority of component studies had to had severe concerns from the JBI checklist), moderate or high heterogeneity (*I* ^2^ > 50%)/inconsistency (deemed present if sensitivity analyses revealed that the removal of one study affected the direction of significance of results), indirectness (the majority of studies in an outcome had to have not reported method of gathering suicides and/or methods of SMI diagnosis), imprecision (defined in this study as total *n* < 10,000), and publication bias (defined as either Egger's *p* = >0.05 in outcomes with >10 studies, or visual evidence of publication bias via funnel plot, see Data S1).

## 3 RESULTS

**FIGURE 1:**
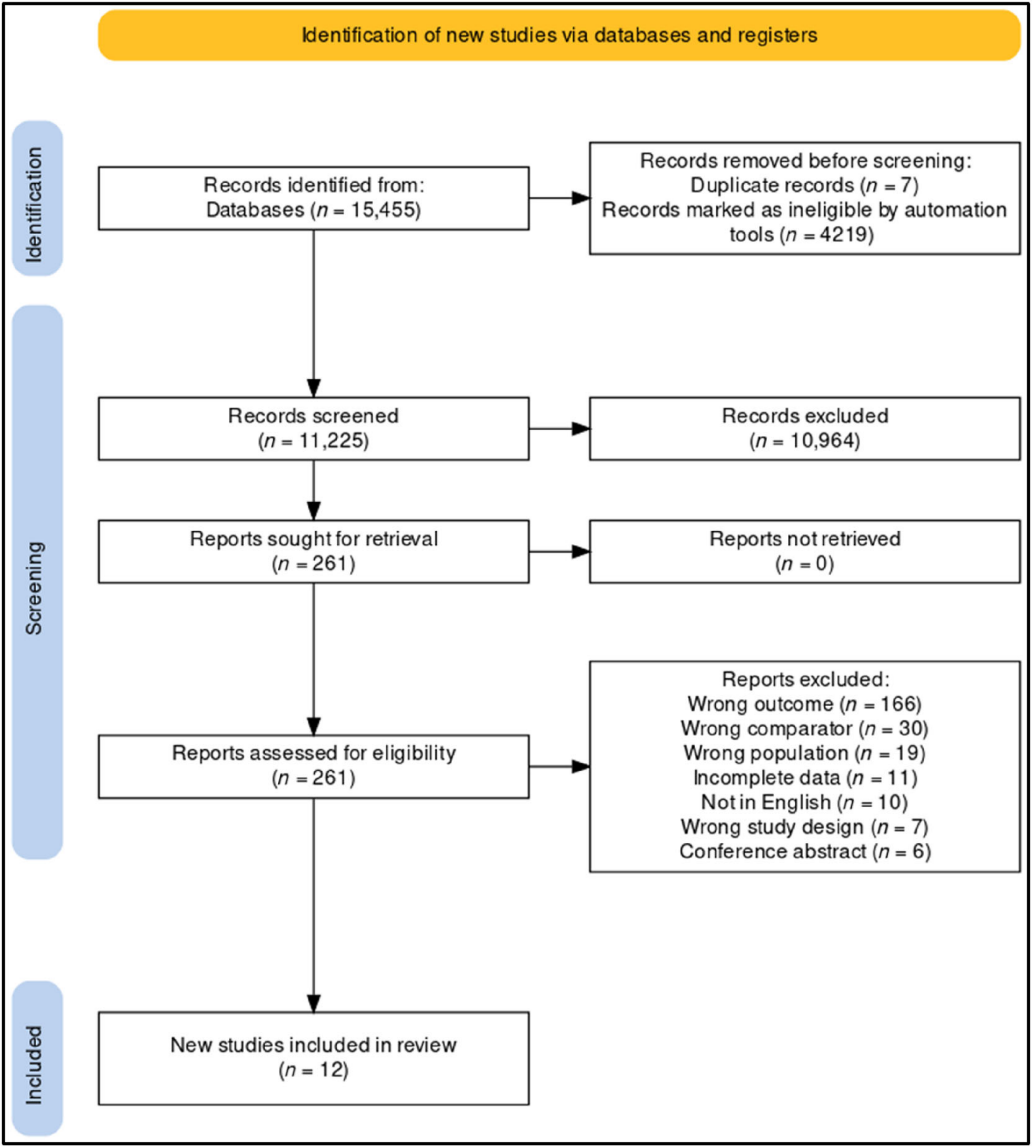
PRISMA flowchart showing included studies.

From the initial search of 11,225 non‐duplicated studies, 12 studies met inclusion criteria, had sufficient data to be used in the meta‐analyses and were included in this review (Figure 1).

Descriptive information on included studies (total *n* = 380,523) can be found in Table 1. Regarding types of SMI that yielded enough studies (*k* = >2) for analysis, all 12 studies examined people with schizophrenia (*n* = 150,475; median *n* per study = 235), eight in major depression (*n* = 137,116, median *n* per study = 5127) and five studies in bipolar disorder (*n* = 92,932; median *n* per study = 6160). Several methods of suicide were reported and were grouped in the following categories: hanging; jumping from heights; fire/burning; drug overdose; gas poisoning; other poisoning (either unspecified or other specified poisoning, such as pesticide); firearms; drowning and cutting. Half of the included studies were from North America, two were from Europe and four were from Asia. Years of data collection ranged from 1972 to 2018. No studies were excluded based on their risk of bias—full scoring information can be found in Table S2. In all outcomes with >10 studies, the Egger's test did not indicate any significant publication bias (see Table 2). For remaining outcomes, funnel plots indicated no clear evidence of publication bias, see Figures S1–S20.

**TABLE 1:** Descriptive information on included studies.

Authors	Country	Study type and cohort information	Mental illness type(s)	Total *n* (*n* SMI cases; *n* no‐SMI control)	Method of data collection	Mean age (SD)	Gender (percentage female)	Mental illness diagnosis method	Suicide method(s)
Black^25^	US	Retrospective cohort; local population (1 US state)	Schizophrenia	68 (18; 50)	Record linkage	NR	NR	ICD‐9 codes	Cutting; drowning; gaseous and non‐gaseous poisoning; hanging; jumping from heights; shooting
Breier and Astrachan^26^	US	Retrospective cohort; local population (local hospital)	Schizophrenia	38 (20; 18)	Chart audit	SMI cases 30.3 (8.2); non‐SMI cases: 43.7 (16.4)	SMI cases 10%; non‐SMI cases: 44%	DSM‐III	Cutting; drowning; fire; gaseous and non‐gaseous poisoning; hanging; jumping from heights; shooting
Chen et al.^9^	Taiwan	Retrospective cohort; national population	Bipolar	5779 (482; 5297)	National Health Insurance data files	46.7 (16.4)	NR	ICD‐9 codes	Charcoal burning; hanging; jumping from heights; non‐gaseous poisoning
			Depression	6192 (895; 5297)		49.3 (16.4)			
			Schizophrenia	6000 (703; 5297)		38.2 (11.8)			
Choi et al.^27^	US	Retrospective cohort; local population (16 US states)	Bipolar	6160 (1030; 5130)	National Violent Death Reporting System	NR	100%	DSM‐IV	Hanging; non‐gaseous poisoning; firearms
			Depression	11,005 (5875; 5130)			100%		
			Schizophrenia	5416 (286; 5130)			100%		
Currie et al.^8^	US	Retrospective cohort; local population (18 US states)	Bipolar	73,496 (7218; 66,278)	National Violent Death Reporting System	NR	23% (total across all SMI)	NR	Suffocation; non‐gaseous poisoning; firearms
			Depression	103,507 (37,229; 66,278)					
			Schizophrenia	68,717 (2439; 66,278)					
Docherty et al.^28^	US	Retrospective cohort; local population (1 US state)	Bipolar	911 (237; 674)	Office of Medical examiner	SMI cases 38 (14) non‐SMI cases 35 (15)	SMI cases 67%; non‐SMI cases: 15%	ICD‐10 codes	Asphyxiation; non‐gaseous poisoning; gunshot
			Depression	1363 (689; 674)		SMI cases 41 (16) non‐SMI cases 35 (15)	SMI cases 36%; non‐SMI cases: 15%		
			Schizophrenia	782 (108; 674)		SMI cases 39 (13) non‐SMI cases 35 (15)	SMI cases 32%; non‐SMI cases: 15%		
Kim et al.^29^	Korea	Retrospective cohort; national population	Bipolar	6586 (401; 6185)	The Korean National Investigations of Suicide Victims	NR	30% total	NR	Drowning; gaseous and non‐gaseous poisoning; hanging; jumping from heights
			Depression	10,723 (4538; 6185)					
			Schizophrenia	6904 (719; 6185)					
Lyu and Zhang^30^	China	Case control; local population (3 Chinese provinces)	Schizophrenia	242 (38; 204)	Disease Control and Prevention and Psychological autopsy	SMI cases 29.0 (5.6) non‐SMI cases 25.2 (6.4)	SMI cases 61%; non‐SMI cases: 53%	ICD‐10 codes	Drowning; gaseous and non‐gaseous poisoning; hanging, jumping from heights; wrist cutting; railways; electronic; suffocation
Nowers and Gunnell^31^	UK	Retrospective cohort; local population (one UK city)	Depression	254 (127; 127)	Coroner	NR	NR	NR	Jumping from heights
			Schizophrenia	254 (127; 127)					
O'Dwyer et al.^32^	Ireland	Retrospective cohort; local population (1 regional hospital)	Depression	10 (5;5)	Post‐mortem, local coroners, police and other	SMI cases 43.6 (20.3) non‐SMI cases 24.8 (19.2)	SMI cases 0%; non‐SMI cases: 0%	NR	Drowning; hanging; non‐gaseous poisoning; gunshot
			Schizophrenia	8 (3;5)		SMI cases 38.0 (19.0) non‐SMI cases 24.8 (19.2)	SMI cases 33%; non‐SMI cases: 0%		
Pan et al.^33^	Taiwan	Retrospective cohort; national popaultion	Schizophrenia	62,046 (3033; 59,013)	Taiwan National Health Insurance Research Database	NR	NR	ICD‐9 codes	Drowning; charcoal burning; hanging; jumping from heights; non‐gaseous poisoning; cutting firearms
Sinyor et al.^34^	Canada	Retrospective cohort; local population (1 Canadian city)	Depression	4062 (153; 3909)	Office of the Chief Coroner	NR	NR	NR	Charcoal burning; gaseous poisoning

Abbreviations: DSM‐III, Diagnostic and Statistical Manual of Mental Disorders, Third Edition; DSM‐IV, DSM, Fourth Edition; ICD‐9, International Statistical Classification of Diseases and Related Health Problems, 9th revision; ICD‐10, 10th revision; NR, not reported.

**TABLE 2:** Meta‐analyses showing odds of suicide in people with severe mental illness compared with people without severe mental illness.

		*k* studies	*n* participants	Meta‐analysis			Heterogeneity	Publication bias	Level of certainty (GRADE)
Outcome				Odds ratio (95% CI)	*p*‐value	Prediction interval	*I* ^2^	Egger's *p*‐value	
Any type of SMI	Jumping from height*	8	83,191	**2.82** **(2.50–3.18)**	**<0.001**	2.11–3.76	52.49	NA	Moderate
	Fire*	4	73,523	**0.56** **(0.41–0.77)**	**<0.001**	0.16–1.94	83.56	NA	Very low
	Firearms*	7	167,930	**0.63** **(0.53–0.75)**	**<0.001**	0.40–1.01	86.14	NA	Very low
	Drowning	6	74,405	1.38 (1.02–1.85)	0.03	0.66–2.88	65.61	NA	NS
	Gas poisoning	6	17,576	0.66 (0.40–1.08)	0.10	0.17–2.59	74.56	NA	NS
	Other poison	5	121,019	0.93 (0.44–1.98)	0.85	0.07–12.74	98.51	NA	NS
	Drug overdose	5	88,972	1.55 (0.99–2.43)	0.06	0.30–8.07	97.88	NA	NS
	Cutting/use of sharp objects	4	62,544	1.05 (0.81–1.36)	0.72	0.81–1.36	0.00	NA	NS
	Hanging	11	210,149	0.83 (0.66–1.04)	0.10	0.39–1.74	97.04	0.28	NS
Schizophrenia	Drowning*	6	69,306	**1.93** **(1.50–2.48)**	**<0.001**	1.09–3.41	34.77	NA	Moderate
	Jumping from height*	8	75,552	**3.38** **(2.08–5.50)**	**<0.001**	0.82–13.85	93.73	NA	Moderate
	Fire	3	68,084	0.46 (0.24–0.90)	0.02	0.00–832.99	91.67	NA	NS
	Hanging	10	15,0221	0.73 (0.49–1.06)	0.01	0.21–2.48	96.08	0.82	NS
	Firearms	7	137,075	0.60 (0.34–1.05)	0.07	0.10–3.54	92.17	NA	NS
	Gas poisoning	4	7252	0.47 (0.13–1.72)	0.25	0.00–77.25	61.82	NA	NS
	Cutting/use of sharp objects	3	62,394	1.04 (0.80–1.34)	0.79	0.26–1.35	0.00	NA	NS
	Drug overdose	4	75,148	1.60 (0.94–2.74)	0.09	0.13–20.18	95.03	NA	NS
	Other poisoning	5	75,065	0.66 (0.31–1.38)	0.27	0.05–9.11	96.79	NA	NS
Bipolar	Jumping from heights*	2	12,365	**3.20** **(2.70–3.78)**	**<0.001**	2.70–3.78	0.00	NA	Moderate
	Drug overdose	3	13,658	2.44 (1.28–4.65)	0.007	0.00–7935.59	92.70	NA	NS
	Other poisoning	2	79,275	2.01 (0.74–5.44)	0.17	0.74–5.44	98.74	NA	NS
	Hanging	5	92,932	0.86 (0.64–1.16)	0.34	0.28–2.69	93.69	NA	NS
Depression	Jumping from heights*	3	17,169	**2.11** **(1.93–2.31)**	**<0.001**	1.93–2.31	0	NA	Moderate
	Firearms*	4	115,885	**0.43** **(0.33–0.56)**	**<0.001**	0.14–1.29	95.16	NA	Very low
	Drug overdose*	3	23,092	**3.11** **(1.53–6.31)**	**0.002**	0.00–26604.93	98.61	NA	Very low
	Other poisoning	3	109,709	1.51 (0.61–3.73)	0.37	0.00–46471.59	98.15	NA	NS
	Fire	2	10,254	0.60 (0.35–1.01)	0.06	0.35–1.01	62.20	NA	NS
	Drowning	2	10,733	0.93 (0.79–1.11)	0.42	0.79–1.11	0.00	NA	NS
	Gas poisoning	2	14,785	0.80 (0.44–1.46)	0.47	0.44–1.46	91.69	NA	NS
	Hanging	6	132,800	0.99 (0.81–1.21)	0.90	0.50	95.37	NA	NS

Abbreviations: CI, confidence interval; NA, not applicable; NS, not significant.

Bold values indicate statistically significant results after multiple correction (*p* = <0.002).

### 3.1 Suicide methods in SMI compared with no‐SMI

#### 3.1.1 Any type of SMI

**FIGURE 2:**
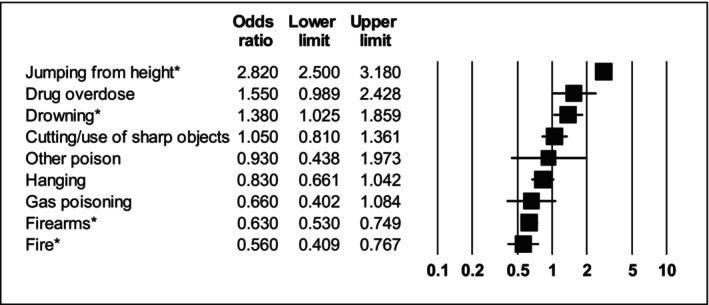
Forest plot showing odds ratios of people who died by suicide by method of suicide in people diagnosed with either major depression, schizophrenia or bipolar disorder compared with the general population. *Indicates statistically significant result.

Compared with people without diagnosed SMI, people with any type of SMI had 2.82 times higher odds of jumping from heights (95% CI: 2.50–3.18; *I* ^2^ = 52.5) as a suicide method. People with SMI also had 1.79 times lower odds of using fire/burning (OR = 0.56, 95% CI: 0.41–0.77; *I* ^2^ = 85.6) and 1.59 times lower odds of using firearms (OR = 0.63, 95% CI: 0.53–0.75; *I* ^2^ = 86.1) than those without SMI although sensitivity analyses indicated that the latter finding was only significant in US studies (see Table S3). All other modes of suicide were not significantly different in people with SMI versus no SMI. The findings are summarised in Table 2 and Figure 2.

#### 3.1.2 Schizophrenia

**FIGURE 3:**
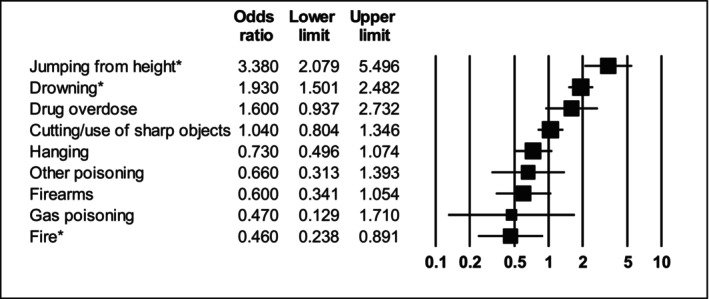
Forest plot showing odds ratios of suicide methods in people with schizophrenia versus people with no severe mental illness. *Indicates statistically significant result.

Compared with people with no previously diagnosed SMI, people with schizophrenia had 3.38 times higher odds of jumping from heights (95% CI: 2.08–5.50; *I* ^2^ = 93.7) and 1.93 times higher odds of drowning (95% CI: 1.50–2.48; *I* ^2^ = 34.8). Although pooled analyses yielded no significant relationship between schizophrenia and suicide by firearms, sensitivity analysis found a significant relationship, with US‐based studies reporting that people with schizophrenia had 2.38 times lower odds of using firearms (OR = 0.42, 95% CI: 0.28–0.63; *I* ^2^ = 79.2—see Table S3). All other suicide methods examined in people with schizophrenia were not significantly different to those without SMI (see Table 2 and Figure 3).

#### 3.1.3 Bipolar disorder

**FIGURE 4:**
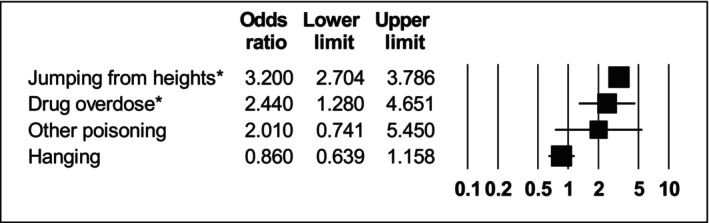
Forest plot showing odds ratios of suicide methods in people with bipolar versus people with no severe mental illness. *Indicates statistically significant result.

Compared with people with no prior SMI diagnosis, people with bipolar disorder had odds of jumping from heights 3.2 times higher (95% CI: 2.70–3.78; *I* ^2^ = 0). All other modes of suicide did not show any statistically significant difference compared with people with no previous SMI diagnoses (see Table 2 and Figure 4).

#### 3.1.4 Major depressive disorder

**FIGURE 5:**
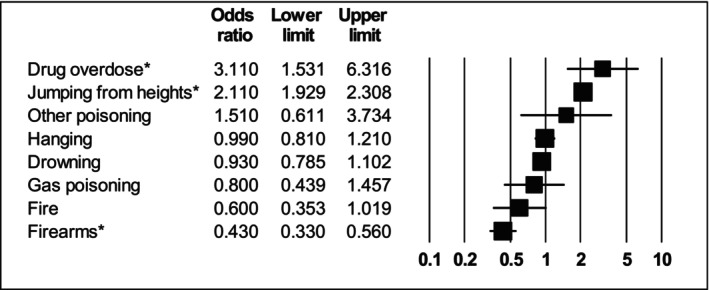
Forest plot showing odds ratios of suicide methods in people with major depression versus people with no severe mental illness. *Indicates statistically significant result.

Compared with people with no prior SMI diagnosis, people with depression had 3.11 times higher odds of drug overdose (OR = 3.11 95% CI: 1.53–6.31; *I* ^2^ = 98.6) and 2.11 times higher odds of dying by jumping from heights (95% CI: 1.93–2.31; *I* ^2^ = 0). However, people with depression odds of suicide by firearms 2.33 times lower (OR = 0.43 95% CI: 0.33–0.56; *I* ^2^ = 95.2; all US studies). There were no statistically significant differences in other suicide methods (see Table 2 and Figure 5).

When suicide methods were dichotomised into violent or non‐violent, there were no significant differences between the effect sizes of violent and non‐violent methods in people with major depression versus people with no SMI after multiple correction (1.73 vs. 0.67; *p* = 0.035). No significant differences were found between violent and non‐violent methods in people with schizophrenia or bipolar disorder (see Table 3).

**TABLE 3:** Differences between odds of violent versus non‐violent methods of suicide in people with versus without severe mental illness.

Outcome	Subgroup	*k* studies	Meta‐analysis		Heterogeneity	*p* value for difference between groups
			Odds ratio (95% CI)	*p*‐value	*I* ^2^	
Schizophrenia	Non‐violent	8	0.93 (0.63–1.37)	0.72	93.96	0.77
	Violent	9	1.01 (0.70–1.45)	0.96	93.19	
Bipolar	Non‐violent	4	1.24 (0.34–4.51)	0.74	99.26	0.63
	Violent	4	0.79 (0.22–2.84)	0.72	99.26	
Depression	Non‐violent	6	1.73 (0.90–3.30)	0.10	98.88	0.035
	Violent	7	0.67 (0.37–1.22)	0.19	98.67	

### 3.2 Prevalence proportions

#### 3.2.1 All SMI

Amongst people with all types of SMI, dying by firearms was the most prevalent (16.24%; 95% CI: 14.02%–21.32%), followed by drug overdose (15.51%; 95% CI: 12.85%–16.35%), and hanging (15.14%; 95%CI: 12.33%–21.74%). Full information can be found in Table 4.

**TABLE 4:** Meta‐analyses showing pooled prevalence rates of suicide methods across people with severe mental illness.

Outcome		*k* studies	Prevalence proportion (95% CI)	*I* ^2^
Any type of SMI	Firearms	14	16.24% (14.02–21.32)	99.9
	Drug overdose	8	15.51% (12.85–16.35)	99.8
	Hanging	20	15.14% (12.33–21.74)	98.8
	Other poisoning	11	14.60% (12.80–18.65)	96
	Jumping from heights	12	11.69% (11.22–12.51)	97.3
	Gaseous poisoning	7	9.47% (0.00–14.40)	98.7
	Drowning	9	8.73% (1.36–11.53)	91.6
	Fire	6	7.65% (6.34–10.70)	83.6
	Cutting/sharp objects	4	0.98% (0.88–1.01)	0
Schizophrenia	Drug overdose	3	16.86% (0.00–20.08)	99
	Other poisoning	6	16.71% (15.68–20.71)	86.5
	Firearms	6	16.13% (15.29–19.16)	99.6
	Hanging	9	14.40% (10.35–35.26)	86.8
	Drowning	6	13.52% (0.00–17.08)	87.8
	Jumping from heights	7	11.84% (10.36–11.89)	98
	Fire	3	7.70% (6.94–11.16)	93.2
	Gas poisoning	4	1.68% (1.47–1.69)	38.7
	Cutting/sharp objects	4	1.16% (1.00–1.89)	0
Bipolar	Firearms	3	17.63% (15.80–22.18)	96.3
	Other poisoning	2	17.46% (14.97–23.64)	90.4
	Hanging	4	17.29% (15.68–21.26)	97.8
	Drug overdose	3	16.72% (0.50–23.27)	99.6
	Jumping from heights	2	14.55% (12.12–15.53)	95.3
	Fire	1	8.30% (7.12–11.20)	0
	Gaseous poisoning	1	4.16% (3.93–4.75)	0
	Drowning	1	3.89% (3.70–4.35)	0
Depression	Gaseous poisoning	2	21.07% (0.00–28.44)	99.7
	Hanging	6	16.23% (13.20–24.32)	99.6
	Firearms	5	16.18% (13.07–24.56)	99.4
	Drug overdose	2	14.09% (0.00–19.61)	100
	Jumping from heights	3	11.69% (10.66–13.46)	95.8
	Other poisoning	3	11.23% (8.29–19.84)	82.8
	Fire	2	7.05% (5.02–13.08)	0
	Drowning	2	2.47% (1.72–4.74)	0

Abbreviation: CI, confidence interval.

#### 3.2.2 Schizophrenia

**FIGURE 6:**
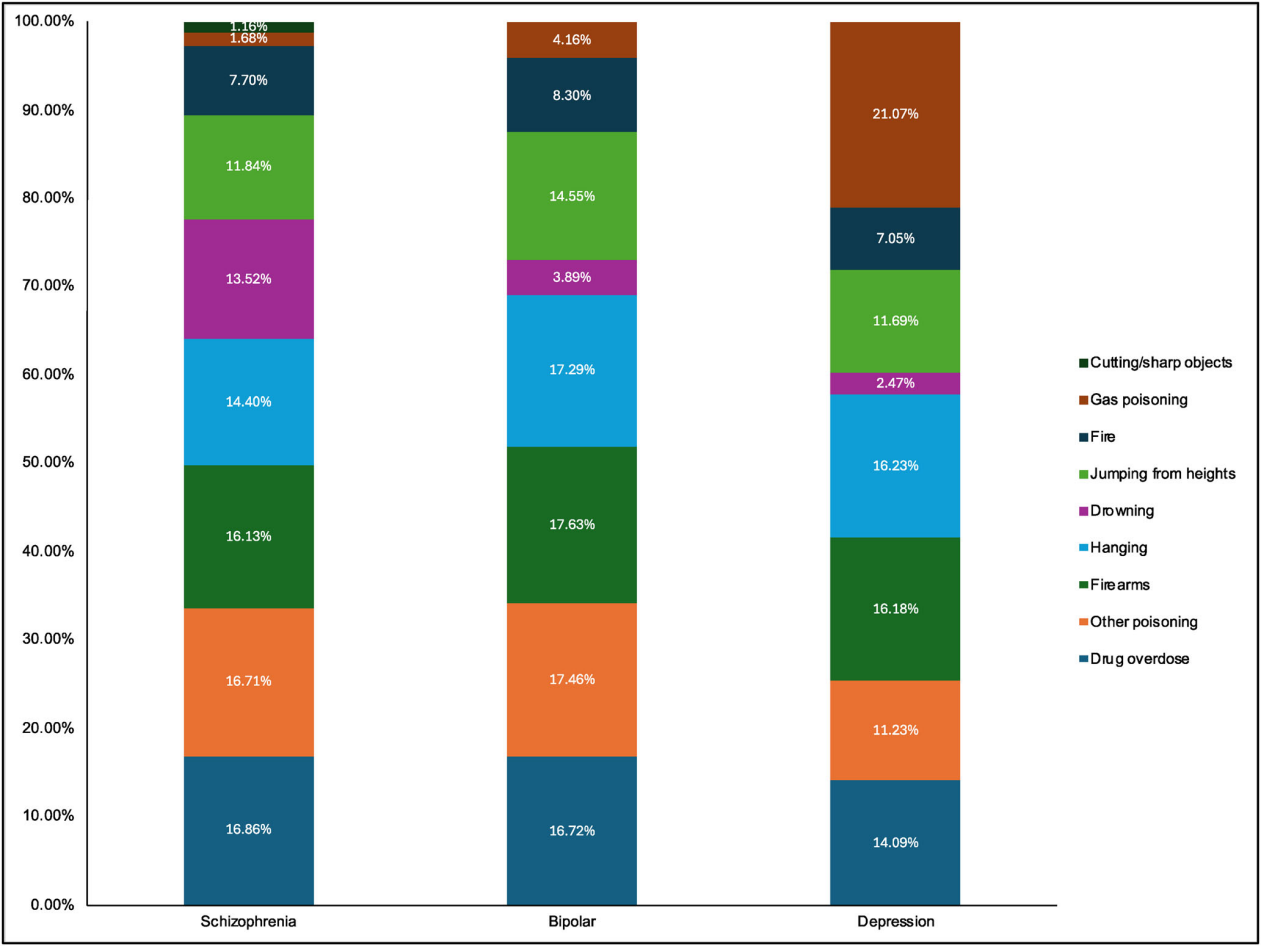
Prevalence proportions showing methods of suicide across schizophrenia, bi‐polar disorder and major depressive disorder.

Amongst people with schizophrenia, dying by drug overdose was the most prevalent (16.86%; 95% CI: 0.00%–20.08%), followed by other poisoning (16.71%; 95% CI: 15.68%–20.71%), and firearms (16.13%; 95%CI: 15.29%–19.16%). Full information can be found in Table 4 and Figure 6.

#### 3.2.3 Bipolar

Amongst people with bipolar, dying by shooting was the most prevalent (17.63%; 95% CI: 15.80%–22.18%), followed by other poisoning (17.46%; 95% CI: 14.97%–23.64%), and hanging (17.29%; 95%CI: 15.68%–21.26%). Full information can be found in Table 4 and Figure 6.

#### 3.2.4 Major depressive disorder

Amongst people with major depressive disorder, dying by gaseous poisoning was the most prevalent (21.07%; 95% CI: 0.00%–28.44%), followed by hanging (16.23%; 95% CI: 13.20%–24.32%), and firearms (16.18%; 95%CI: 13.07%–24.56%). Full information can be found in Table 4 and Figure 6.

#### 3.2.5 Sub‐group and sensitivity analyses

Sensitivity analyses using the one‐study removed method indicated that the removal of any one study did not change the significance or direction of effect sizes. Exploratory meta‐regression analyses found that cohort mean age and gender did not significantly moderate significant effect sizes across all SMI and schizophrenia subgroups (*k* studies was too low to run exploratory meta‐regressions for bi‐polar and depression sub‐groups). Suggestive evidence was found showing that the odds of jumping from heights increased by 3% (95% CI: 0%–6%; *p* = 0.03) per increasing year of publication in people with all SMI. Furthermore, suggestive evidence was found that the odds of jumping from heights increased by 5% (95% CI: 0%–6%; *p* = 0.03) per increasing year of publication in people with schizophrenia, see Table S5 for more information. Regarding prevalence proportions, suggestive evidence was found that, in people with schizophrenia and dying of other poisoning, increasing age lowered the prevalence (*p* = <0.001), and if more males were included the prevalence increased (<0.001). Moreover, the prevalence of dying by drug overdose decreased per increasing year of publication (*p* = <0.001), see Table S6.

Sub‐group analyses by geographical location showed that only studies from Asia yielded significant results across all significant outcomes (sub‐group analysis by bi‐polar was not possible due to a lack of studies), see Table S7 for more information.

#### 3.2.6 Certainty of evidence and risk of bias

Although some studies had moderate concerns regarding risk of bias (see Table S2), no studies were deemed to have high risk of bias, and therefore no studies were downgraded due to this. Furthermore, no outcome was downgraded due to imprecision or indirectness. Three outcomes were classified as having a moderate degree of certainty (jumping from heights in any type of SMI and major depression, and drowning in schizophrenia), with all other remaining significant outcomes being classified as very low degree of certainty, predominantly because of high heterogeneity (several outcomes examined had moderate to high heterogeneity) (*I* ^2^ > 0%). Full information on how significant findings were classified can be found in Table S4.

## 4 DISCUSSION

Combining data from 380,523 individuals across 12 studies, this systematic review examined if there are statistically significant differences between suicide methods of people with SMI compared with those without SMI, and to explore differences in suicide methods across different types of SMIs. The results showed that people with any type of SMI had almost three times higher odds of jumping from heights compared with those without SMI, with a moderate degree of heterogeneity. By contrast, people with SMI had almost 1.8 times lower odds of use fire, smoke or burning and 1.6 times lower odds of using firearms as a suicide method compared with those without SMI, with high degrees of heterogeneity. Results also found that the prevalence of suicide methods differed according to type of SMI.

When stratified according to the type of SMI, people with schizophrenia had over threefold higher odds of suicide by jumping from heights, with similar results being found for people with bipolar disorder (over three times higher odds) and major depression (over two times higher odds). Interestingly, heterogeneity for the jumping from heights outcomes was only high for schizophrenia, possibly highlighting the heterogeneous nature of the illness. People with schizophrenia also had odds almost two times higher of suicide by drowning when compared with people with no diagnosed SMI, with low heterogeneity. Although differences in heterogeneity across schizophrenia outcomes could reflect the heterogeneous nature of the illness, it could also be because of differences in reporting methods and/or cultures across outcomes. People with depression had over three times higher odds of suicide by drug overdose, however heterogeneity for this outcome was very high, possibly because of differences in definitions of ‘drug overdose’ across a limited number of studies. Of note, suicide by hanging was not statistically significantly different between people with any SMI compared with people with no SMI. No statistically significant differences were found between violent and non‐violent modes of suicide.

Somewhat surprisingly, the current review found that people with depression were less likely to die by suicide using firearms compared with those without SMI, although this was only found in studies from the US, and had high heterogeneity. It should be noted that firearms are more accessible in the US compared with most other countries in the world; the average number of firearms possessions per 100 people in the US is 120.5, compared with 34.7 in Canada, 14.5 in Australia, and 4.6 in the UK.^35^ It may be postulated that this is because people with SMI are less likely to have access to firearms due to legislative restriction or be in occupations where firearms are more easily available (e.g., armed forces and police), this is a finding that warrants further exploration. Our results also suggest that the method of suicide in SMI are not more or less violent than people with no diagnosed SMI. These results, however, should be taken with caution, as it is likely that the non‐violent group lacked statistical power. Future research is warranted to see if this relationship exists in suicide attempts.

These findings, as well as the differences in prevalence rates by type of SMI, support the hypothesis that people with diagnosed SMI have significantly different patterns of suicide method use compared with those without diagnosed SMI, and have different patterns of suicide by type of SMI. The increased risk of suicide by jumping from heights may represent increased impulsivity seen in people with all three of the SMIs included in this review.^36^, ^37^, ^38^ The increased risk for jumping from heights and drowning amongst those with schizophrenia may be due to severe psychotic phenomenology (e.g., command hallucinations), which may not be present in bi‐polar or major depression, however this hypothesis needs further examination in primary studies. The association between schizophrenia and drowning is generally in agreement with the existing literature.^39^, ^40^ For depression, the increased risk of suicide by drug overdose requires further investigation, as previous studies have largely found no differences between the prevalence of substance use/abuse across schizophrenia, bipolar and major depression.^41^, ^42^ Further reliable prevalence studies are required to confirm or refute these findings, first to determine the sources of heterogeneity in the extant literature, and second to assess whether drugs in these populations may be amenable to means restriction. It should also be emphasised, moreover, that drug overdoses include both prescription and un‐prescribed/recreational drugs. Nevertheless, this result concurs with the literature more broadly—for example, one meta‐analysis found that depression was significantly associated with non‐fatal drug overdoses.^43^

The findings of this review have important public health and clinical implications. First, given the disproportionally high representation of people with SMI who die by suicide by jumping from heights, it is worthwhile considering barrier installation at suicide hotspots, which have been shown to be effective across several studies. For example, a time series study of suicide prevention at the Story Bridge in Brisbane, Australia^44^ provides a successful example of such public health intervention. Although installing Lifeline phones, signs and surveillance cameras did not reduce the number of suicides by jumping from the bridge, there was a reduction in suicides after barrier installation. Several systematic reviews have also established the dramatic reductions in suicide by jumping at specific sites after the installation of barriers.^45^, ^46^, ^47^ The increased risk of suicide using drug overdose amongst people with depression emphasises the importance of prescribers considering the results of this study when conducting a risk/benefit analysis on the prescribing of high lethality psychotropics, reducing the dispensing quantity (e.g., less than 1 week's supply), potentially limiting the package size of over‐the counter medications, and real‐time monitoring of prescription dispensing. Although our results regarding drug overdoses could be relating to recreational drugs, research is also warranted to determine more specifically how psychotropic medications are prescribed to this population (e.g., what quantity they are given at any given time and at what stage of treatment). A monitoring system (similar to ones already in existence for controlled/authorised medicines in many jurisdictions) may be useful to allow prescribers to monitor the quantity of medications given to their more at‐risk patients. The results of this study also provides evidence that lethal means counselling services should consider SMI diagnoses, including the potential development of safety plan templates targeted towards psychiatric diagnostic groups, which may improve the effectiveness of these programmes.

The results of this review should be considered within its limitations. First, although there was a large geographical spread regarding the sources of data, almost all the studies collected data from high income counties, with five studies from the US, two from the UK and Taiwan respectively, and one respectively from South Korea, Ireland and Canada—the only low‐middle income country was one study from China. Considering that we were only able to obtain 12 studies, these limitations preclude generalisability of results. Related to this, it is important to acknowledge that suicide methods are often culture and country specific, and are influenced by the political and economic factors as well as the time in history.^7^ Although we did run a sub‐group analysis that showed significant results across only Asian studies, this was likely due to low statistical power across the other sub‐groups. Furthermore, we found suggestive evidence of time trends regarding the prevalence of drug overdoses in schizophrenia, however a lack of statistical power precludes conclusions to be made. Future primary studies are urgently needed to examine this further.

Second, because unadjusted data were used in analyses (due to unavailability of sub‐grouped demographic information), relevant factors, such as age, sex, sub‐types/phases of SMI and other comorbidities, such as substance abuse could not be controlled for. Future primary studies should include relevant demographic information by sub‐group so that meaningful descriptive analyses and meta‐regressions can be conducted to see if these variables significantly influence results. Third, although we examined the group differences between those with diagnoses of SMI and those without, it is highly possible that the comparison group may have had a large proportion of people with undiagnosed SMI, or other types of disorder, such as substance abuse. A further limitation is the low k studies found for this analysis. As such, the findings of this study should be considered with caution. Lastly, the methods of reporting types of suicide differ between countries, and between time, which could cause heterogeneity. Given heterogeneity was high for most outcomes, which (due to low *k* studies) could not be further explored, our findings should be treated with caution, even though we used random effects models.

To conclude people with different SMIs have different patterns of suicide method use compared with those without SMI, with notably higher rates of jumping from heights across all three types of SMI. Furthermore, the prevalence rates of suicide methods differ across different types of SMI. Our findings should inform future initiatives, which can help build more nuanced and targeted suicide prevention strategies both at the public health and clinical levels for people living with SMI. Specifically, this research has the potential to inform lethal means counselling practices in this population.

## FUNDING INFORMATION

DS, in part, was funded by an NHMRC Emerging Leadership Fellowship (GNT1194635). All other authors received no financial support from any funding agency, commercial or not‐for‐profit sectors.

## CONFLICT OF INTEREST STATEMENT

The authors declare no conflict of interest.

### PEER REVIEW

The peer review history for this article is available at https://www.webofscience.com/api/gateway/wos/peer‐review/10.1111/acps.13759.

## Supporting information


**Data S1.** Supporting Information.

## Data Availability

All data used in this study are from publicly available sources—no new data was created in this study.
