# Supplementary material for: Suicide methods and severe mental illness: A systematic review and meta‐analysis
Source: Acta Psychiatr Scand. 2024 Oct 1;151(4):467–84. doi: 10.1111/acps.13759 (PMC11884913; doi:10.1111/acps.13759)
Supplement: Supplementary file 1 — Data S1. Supporting Information. [file ACPS-151-467-s001.docx]

**Supplementary Table 1**: Search strategy

| Database | Platform | Suicide method terms | Mental disorder terms |
| --- | --- | --- | --- |
| CINAHL and PsycINFO | EBSCOhost | *Free-text terms* | *Free-text terms* |
|  |  | TI parasuicid* OR AB parasuicid*  OR TI para-suicid* OR AB para-suicid*  OR TI “self harm*” OR AB “self harm*”  OR TI “self inflicted injur*” OR AB “self inflicted injur*”  OR TI “self injur*” OR AB “self injur*”  OR TI “self mutilat*” OR AB “self mutilat*”  OR TI “self poison*” OR AB “self poison*”  OR TI “self stab*” OR AB “self stab*”  OR TI “suicide method” OR AB “suicide method”  parasuicide[Tiab] OR parasuicides[Tiab] OR para-suicide[Tiab] OR para-suicides[Tiab] OR "self harm"[Tiab] OR "self harm"[Tiab] OR "self harmed"[Tiab] OR "self inflicted injury"[Tiab] OR "self inflicted injuries"[Tiab] OR "self injury"[Tiab] OR "self injuries"[Tiab] OR "self mutilation"[Tiab] OR TI "self poison*"[Tiab] OR "self poison*"[Tiab] OR "self stabbing"[Tiab] OR "suicide method"[Tiab] OR OR[Tiab] OR OR[Tiab] OR OR[Tiab] OR OR[Tiab] | TI anorexi* OR AB anorexi*  OR (TI anxiety OR AB anxiety) OR N3 (TI disorder* OR AB disorder* OR TI diagnos* OR AB diagnos*) OR MH "anxiety+"  OR TI bulimi* OR AB bulimi*  OR TI bipolar OR AB bipolar OR MH bipolar disorder+  OR (TI depress* OR AB depress*) N3 (TI disorder* OR AB disorder* OR TI diagnos* OR AB diagnos*)  MH depression+  OR TI dysthymi* OR AB dysthymi*  OR TI “eating disorder*” OR AB “eating disorder*” OR MH eating disorders+  OR TI “personality disorder*” OR AB “personality disorder*” OR MH personality disorders+  OR TI “psychiatric diagnos*” OR AB “psychiatric diagnos*”  OR TI “psychiatric disorder*” OR AB “psychiatric disorder*”  OR TI psychopathology OR AB psychopathology OR MH psychopathology  OR (TI psychotic OR AB psychotic OR TI psychosis OR AB psychosis OR MH psychotic disorders+  OR TI schizo* OR AB schizo*  OR TI “use disorder*” OR AB “use disorder*” OR MH substance use disorder |
|  |  | *Controlled vocabulary - CINAHL Subject Headings* | *Controlled vocabulary - CINAHL Subject Headings* |
|  |  | MM "Suicide+"  OR MM "Suicide, Attempted" | MM "Mental Disorders+"  OR MM "Mental Disorders, Chronic"  OR MM "Organic Mental Disorders, Substance-Induced+"  OR (MM "Organic Mental Disorders, Psychotic+")  OR (MM "Organic Mental Disorders+")  OR (MM "Mental Disorders Diagnosed in Childhood+")  OR (MM "Behavioral and Mental Disorders+") |
| PubMed and EMBASE | PubMed and EMBASE | *Free-text terms* | *Free-text terms* |
|  |  | asphyxia*[tiab]  automobile*[tiab]  barbit*[tiab]  “blunt object*”[tiab]  bridge*[tiab]  burning[tiab]  car[tiab]  “car park*”[tiab]  carpark*[tiab]  cars[tiab]  “carbon monoxide”[tiab]  charcoal[tiab]  cliff*[tiab]  crashing*[tiab]  cutting[tiab]  drown*[tiab]  emission*[tiab]  “exhaust fume*”[tiab]  “explo*”[tiab]  fire*[tiab]  flame*[tiab]  gun[tiab]  guns[tiab]  handgun*[tiab]  “hand-gun*”[tiab]  hand-gun*[tiab]  high-rise*[tiab]  "high rise*”[tiab]  insecticide[tiab]  gas[tiab]  gases[tiab]  hanging[tiab]  “hot object*”[tiab]  “hot vap*”[tiab]  “hot vapour*”[tiab]  jump*[tiab]  knife[tiab]  knives[tiab]  leap*[tiab]  lying[tiab]  metro[tiab]  OR organophosp*[tiab]  “over dos*”[tiab]  overdos*[tiab]  over-dos*[tiab]  means[tiab]  “mot vehicle exhaust”[tiab]  parasuicid*[tiab]  para-suicid*[tiab]  pesticide[tiab]  piercing[tiab]  poison*[tiab]  railway*[tiab]  railroad*[tiab]  rifle*[tiab]  self-harm*[tiab]  “self harm*”[tiab]  self-poison*[tiab]  “self poison*”[tiab]  self-stab*[tiab]  “self stab*”[tiab]  “sharp object*”  “shot gun*”[tiab]  “shot-gun*”[tiab]  “shotgun*”[tiab]  “smoke”[tiab]  “steam”[tiab]  “submers*”[tiab]  subway[tiab]  “sub way”[tiab]  strang*[tiab]  suffocat*[tiab]  “suicide”[tiab]  “tall building*”[tiab]  underground[tiab]  vehicle*[tiab] | anorexi*[tiab]  anxiety[tiab]  bulimi*[tiab]  “autism*”[tiab]  bipolar[tiab]  depress*[tiab]  diagnos*[tiab]  dysthymi*[tiab]  disorder*[tiab]  mental[tiab]  psychiatric[tiab]  psychopathology[tiab]  psychot*[tiab]  schizo*[tiab]  “use disorder”[tiab] |
| PubMed only | PubMed | MeSH | MeSH |
|  |  | (((((("Suicide"[Mesh])  OR ( "Smoke Inhalation Injury"[Mesh]  OR "Incineration"[Mesh] ))  OR "Self Mutilation"[Mesh])  OR "Drowning"[Mesh])  OR "Poisoning"[Mesh])  OR ( "Firearms"[Mesh]  OR "Gun Violence"[Mesh] ))  OR "Asphyxia"[Mesh] | ("Mental Disorders"[Mesh]) OR  "Substance-Related Disorders"[Mesh] |

Notes. We used search terms for suicide methods from an ongoing generic protocol for a Cochrane Review of means restriction for the prevention of suicide^1^ and codes *X60-X84 Intentional self-harm* from the *International Statistical Classification of Diseases and Related Health Problems 10th Revision*.^2^ We used a recent Global Burden of Disease study^3^ and an analytic survey of optimal search strategies for identifying mental health content in MEDLINE^4^ to develop search terms for mental disorders and substance use disorders. We also used five known relevant studies^5–10^ to identify additional search terms and confirm that our searches would locate these studies.

**References**

1 John A, Hawton K, Okolie C, Dennis M, Price SF, Lloyd K. Means restriction for the prevention of suicide: generic protocol. *Cochrane Database Syst Rev* 2018; **2018**: CD012995.

2 ICD-10 Version:2010. https://icd.who.int/browse10/2010/en (accessed Aug 4, 2022).

3 Castelpietra G, Knudsen AKS, Agardh EE, *et al.* The burden of mental disorders, substance use disorders and self-harm among young people in Europe, 1990–2019: Findings from the Global Burden of Disease Study 2019. *Lancet Reg Health Eur* 2022; **16**: 100341.

4 Wilczynski NL, Haynes RB, Team Hedges. Optimal search strategies for identifying mental health content in MEDLINE: an analytic survey. *Annals of General Psychiatry* 2006; **5**: 4.

5 Huisman A, van Houwelingen CAJ, Kerkhof AJFM. Psychopathology and suicide method in mental health care. *Journal of Affective Disorders* 2010; **121**: 94–9.

6 Chen Y-Y, Lee M-B, Chang C-M, Liao S-C. Methods of suicide in different psychiatric diagnostic groups. *J Affect Disord* 2009; **118**: 196–200.

7 Lopez-Morinigo J-D, Fernandes AC, Chang C-K, *et al.* Suicide completion in secondary mental healthcare: a comparison study between schizophrenia spectrum disorders and all other diagnoses. *BMC Psychiatry* 2014; **14**: 213.

8 Kimura R, Ikeda S, Kumazaki H, Yanagida M, Matsunaga H. Comparison of the clinical features of suicide attempters by jumping from a height and those by self-stabbing in Japan. *J Affect Disord* 2013; **150**: 695–8.

9 De Leo D, Klieve H. Communication of suicide intent by schizophrenic subjects: data from the Queensland Suicide Register. *Int J Ment Health Syst* 2007; **1**: 6.

10 Kõlves K, McDonough M, Crompton D, de Leo D. Choice of a suicide method: Trends and characteristics. *Psychiatry Research* 2018; **260**: 67–74.

**Supplementary Table 2**: Risk of Bias scoring

| **Authors** | **Was the sample frame appropriate to address the target population?** | **Were study participants sampled in an appropriate way?** | **Was the sample size adequate?** | **Were the study subjects and the setting described in detail?** | **Was the data analysis conducted with sufficient coverage of the identified sample?** | **Were valid methods used for the identification of the condition?** | **Was the condition measured in a standard, reliable way for all participants?** | **Was there appropriate statistical analysis?** | **Was the response rate adequate, and if not, was the low response rate managed appropriately?** |
| --- | --- | --- | --- | --- | --- | --- | --- | --- | --- |
| Black (8) | No | No | No | Yes | Yes | Yes | Yes | Yes | NA |
| Breier and Astrachan (7) | No | No | No | Yes | No | Yes | Yes | No | NA |
| Chen et al. (23) | Yes | Yes | Yes | Yes | Yes | No | No | Yes | Yes |
| Choi et al. (24) | No | No | Yes | Yes | Yes | Yes | Yes | Yes | Yes |
| Currie et al. (6) | Yes | Yes | Yes | Yes | Yes | Yes | Yes | Yes | Yes |
| Docherty et al. (25) | Yes | Yes | Yes | Yes | Yes | Yes | Yes | Yes | Yes |
| Kim et al. (26) | Yes | Yes | Yes | Yes | Yes | Yes | Yes | Yes | Yes |
| Lyu and Zhang (28) | No | Yes | No | Yes | Yes | Yes | Yes | Yes | NA |
| Nowers and Gunnell (29) | No | No | No | Yes | Yes | Yes | Yes | Yes | NA |
| O'Dwyer et al. (30) | No | No | No | Yes | Yes | Yes | Yes | NA | NA |
| Pan et al. (31) | Yes | Yes | Yes | Yes | Yes | Yes | Yes | Yes | Yes |
| Sinyor et al. (32) | No | No | No | Yes | Yes | Yes | Yes | Yes | NA |

**Supplementary Table 3:** Meta-analysis showing odds of completed suicides by method of firearms in people with severe mental illness compared to people without severe mental illness comparing USA to non-USA studies

| **Outcome** | | ***k* studies** | **Meta-analysis** | | | **Heterogeneity** | **Difference between groups** |
| --- | --- | --- | --- | --- | --- | --- | --- |
|  |  |  | **Odds ratio**  **(95% CI)** | **p-value** | **Prediction interval** | **I^2^** |  |
| Any type of SMI | **USA** | 5 | **0.53**  **(0.48-0.59)** | **<0.001** | 0.38-0.74 | 72.13 | <0.001 |
|  | **Non-USA** | 2 | **1.26**  **(0.91-1.74)** | **0.17** | 0.91-1.74 | 0.00 |  |
| Schizophrenia | **USA** | 5 | **0.42**  **(0.28-0.63)** | **<0.001** | 0.11-1.58 | 79.16 | <0.001 |
|  | **Non-USA** | 2 | **1.26**  **(0.91-1.74)** | **0.16** | 0.91-1.74 | 0.00 |  |
| Depression | **USA** | 3 | **0.43**  **(0.33-0.56)** | **<0.001** | 0.02-11.37 | 96.77 | 0.59 |
|  | **Non-USA** | 1 | **1.00**  **(0.05-22.18)** | **1.00** | 0.05-22.18 | 0.00 |  |

Note there were no non-USA studies in bi-polar

**Supplementary Table 4**: Grading of significant outcomes

| Outcomes | | Large effect size^1^ | Risk of bias^2^ | Heterogeneity/inconsistency^2^ | Indirectness^2^ | Imprecision^2^ | Publication bias^2^ | Final GRADE classification |
| --- | --- | --- | --- | --- | --- | --- | --- | --- |
| Any type of SMI | Jumping from height* | Yes | Some concerns | No | No | No | No | Moderate |
|  | Fire* | No | Some concerns | Yes (high heterogeneity) | No | No | No | Very Low |
|  | Firearms* | No | Some concerns | Yes (high heterogeneity) | No | No | No | Very Low |
| Schizophrenia | Drowning* | Yes | Some concerns | No | No | No | No | Moderate |
|  | Jumping from height* | No | Some concerns | Yes (high heterogeneity) | No | No | No | Very Low |
|  | Fire* | No | Some concerns | Yes (high heterogeneity) | No | No | No | Very Low |
| Depression | Jumping from heights* | Yes | Some concerns | No | No | No | No | Moderate |
|  | Firearms* | No | Some concerns | Yes (high heterogeneity) | No | No | No | Very Low |

All studies started with ‘low classification’; ^1^=if present, outcome was upgraded; ^2^=if present, outcomes was downgraded

**Supplementary Table 5**: Exploratory meta-regression results by mean age, gender (percentage female), and year of publication across significant outcomes

|  |  | Age | | | Gender | | | Date of publication | | |
| --- | --- | --- | --- | --- | --- | --- | --- | --- | --- | --- |
|  |  | *k* studies | Log odds  (95% CI) | p-value | *k* studies | Log odds  (95% CI) | p-value | *k* studies | Log odds  (95% CI) | p-value |
| Any SMI | Jumping from heights | 2 | NA | NA | 8 | 1.31  (-2.20; 7.81) | 0.69 | 12 | 0.03  (0.00; 0.06) | 0.03 |
|  | Fire | 1 | NA | NA | 4 | 12.07  (-12.19; 36.32) | 0.33 | 6 | 0.03  (-0.02; 0.07) | 0.25 |
|  | Firearms | 11 | -0.06  (-0.15; 0.03) | 0.21 | 14 | -0.99  (-3.09; 1.11) | 0.36 | 16 | -0.02  (-0.07; 0.03) | 0.42 |
| Schizophrenia | Drowning | 3 | 0.02  (-0.15; 0.19) | 0.83 | 4 | 0.76  (-3.91; 5.43) | 0.75 | 6 | -0.00  (-0.05; 0.05) | 0.86 |
|  | Jumping from heights | 2 | NA | NA | 4 | 2.62  (-1.60; 6.85) | 0.22 | 7 | 0.05  (0.01; 0.10) | 0.03 |
| Bi-polar | Not enough data to run exploratory meta-regressions | | | | | | | | | |
| Depression | Not enough data to run exploratory meta-regressions | | | | | | | | | |

NA = not enough studies to run analysis

**Supplementary Table 6**: Exploratory meta-regression results by mean age, gender (percentage female), and year of publication across prevalent proportions

|  |  | Age | | | Gender | | | Date of publication | | |
| --- | --- | --- | --- | --- | --- | --- | --- | --- | --- | --- |
|  |  | *k* studies | Beta-coefficient  (95% CI) | p-value | *k* studies | Beta-coefficient  (95% CI) | p-value | *k* studies | Beta-coefficient  (95% CI) | p-value |
| Schizophrenia | Drug overdose | NA | NA | NA | NA | NA | NA | 3 | -0.14  (-0.20; -0.09) | <0.001 |
|  | Other poisoning | 3 | -0.04  (-0.06; -0.03) | <0.001 | 5 | 1.31  (0.72; 1.91) | <0.001 | 6 | 0.01  (0.00; 0.02) | 0.15 |
|  | Firearms | 3 | 0.00  (-0.03; 0.03) | 0.84 | 4 | -0.03  (-0.54; 0.48) | 0.91 | 6 | 0.00  (-0.01; 0.01) | 0.43 |
|  | Hanging | 4 | 0.00  (0.00; 0.01) | 0.16 | 4 | 0.13  (-0.04; 0.30) | 0.15 | 9 | 0.00  (0.00; 0.01) | 0.16 |
|  | Drowning | 3 | 0.04  (-0.04; 0.12) | 0.29 | 4 | -1.59  (-3.71; 0.54) | 0.14 | 6 | 0.00  (-0.02; 0.01) | 0.63 |
|  | Jumping from heights | NA | NA | NA | 4 | -0.68  (-2.65; 1.30) | 0.50 | 7 | 0.01  (0.00; 0.02) | 0.01 |
|  | Fire | NA | NA | NA | NA | NA | NA | 3 | 0.00  (0.00; 0.01) | 0.01 |
|  | Gas poisoning | NA | NA | NA | 3 | -0.13 | 0.14 | 4 | 0.00  (0.00; 0.00) | 0.34 |
|  | Cutting/sharp objects | NA | NA | NA | NA | NA | NA | 4 | 0.00  (0.00; 0.00) | 0.33 |
| Bi-polar | Not enough data to run exploratory meta-regressions | | | | | | | | | |
| Depression | Not enough data to run exploratory meta-regressions | | | | | | | | | |

NA = not enough studies to run analysis

**Supplementary Table 7**: Sub-group analysis by geographical location

|  | | | *k* studies | OR  (95% CI) | p-value | I^2^ |
| --- | --- | --- | --- | --- | --- | --- |
| Any SMI | Jumping from heights | North America | 2 | 0.56  (0.08- 4.01) | 0.56 | 0.00% |
|  |  | Asia | 8 | 3.37  (2.50-4.54) | <0.001 | 95.83% |
|  |  | Europe | 2 | 1.56  (0.99-2.45) | 0.05 | 0.00% |
|  | Fire | North America | 2 | 0.91  (0.48-1.71) | 0.76 | 0.00% |
|  |  | Asia | 4 | 0.46  (0.35-0.62) | <0.001 | 88.53% |
|  | Firearms | North America | 13 | 0.76  (0.37-1.56) | 0.46 | 99.84% |
|  |  | Asia | 1 | 1.27  (0.92 – 1.76) | 0.15 | 0.00% |
|  |  | Europe | 2 | 0.69  (0.07-1.56) | 0.75 | 0.00% |
|  | Drowning | North America | 2 | 1.14  (0.16-7.90) | 0.90 | 0.00% |
|  |  | Asia | 5 | 1.55  (1.07-2.23) | 0.02 | 90.66% |
|  |  | Europe | 2 | 1.67  (0.07; 40.51) | 0.75 | 52.53% |
|  | Gas poisoning | North America | 3 | 1.10  (0.80-1.51) | 0.55 | 0.00% |
|  |  | Asia | 4 | 0.41  (0.21-0.81) | 0.01 | 90.77% |
|  | Other poisoning | North America | 5 | 1.42  (0.61-3.31) | 0.42 | 99.80% |
|  |  | Asia | 4 | 0.77  (0.46-1.28) | 0.31 | 94.19% |
|  |  | Europe | 2 | 2.60  (0.19-63.53) | 0.48 | 0.00% |
|  | Drug overdose | North America | 4 | 2.41  (1.54-3.78) | <0.001 | 96.58% |
|  |  | Asia | 4 | 1.07  (0.88-1.31) | 0.50 | 80.03% |
|  | Cutting/sharp objects | North America | 2 | 0.51  (0.05-5.05) | 0.56 | 0.00% |
|  |  | Asia | 2 | 1.05  (0.81-1.37) | 0.70 | 0.00% |
|  | Hanging | North America | 11 | 1.06  (0.96-1.17) | 0.24 | 82.69% |
|  |  | Asia | 10 | 0.69  (0.49-0.97) | 0.03 | 97.48% |
|  |  | Europe | 2 | 0.56  (0.07-4.25) | 0.58 | 0.00% |
| Schizophrenia | Jumping from heights | North America | 2 | 0.56  (0.08-4.01) | 0.56 | 0.00% |
|  |  | Asia | 4 | 4.57  (2.92-7.15) | <0.001 | 95.04% |
|  |  | Europe | 1 | 1.20  (0.52-2.80) | 0.67 | 0.00% |
|  | Fire | North America | 1 | 2.85  (0.11-74.38) | 0.53 | 0.00% |
|  |  | Asia | 2 | 0.43  (0.22-0.85) | 0.02 | 95.63% |
|  | Firearms | North America | 4 | 0.71  (0.25-2.05) | 0.53 | 96.54 |
|  |  | Europe | 1 | 0.43  (0.01-14.08) | 0.63 | 0.00% |
|  |  | Asia | 1 | 1.27  (0.92-1.76) | 0.15 | 0.00% |
|  | Drowning | North America | 2 | 1.14  (0.16-7.90) | 0.90 | 0.00% |
|  |  | Asia | 3 | 1.96  (1.45-2.65) | <0.001 | 67.52% |
|  |  | Europe | 1 | 9.80  (0.33-287.72) | 0.19 | 0.00% |
|  | Gas poisoning | North America | 2 | 0.93  (0.26-3.31) | 0.91 | 0.00% |
|  |  | Asia | 2 | 0.32  (0.05-2.15) | 0.24 | 0.00% |
|  | Other poisoning | North America | 3 | 0.73  (0.19-2.77) | 0.64 | 79.69% |
|  |  | Asia | 2 | 0.51  (0.17-1.49) | 0.22 | 87.41% |
|  |  | Europe | 1 | 0.57  (0.03-98.96) | 0.83 | 0.00% |
|  | Drug overdose | North America | 1 | 1.71  (1.34-2.18) | <0.001 | 0.00% |
|  |  | Asia | 2 | 0.90  (0.82-0.99) | 0.03 | 0.00% |
|  | Cutting/sharp objects | North America | 2 | 0.51  (0.05-5.05) | 0.56 | 0.00% |
|  |  | Asia | 2 | 1.05  (0.81-1.38) | 0.71 | 0.00% |
|  | Hanging | North America | 4 | 1.05  (0.94-1.18) | 0.37 | 3.84% |
|  |  | Asia | 4 | 0.54  (0.28-1.04) | 0.07 | 98.00% |
|  |  | Europe | 1 | 0.20  (0.01-5.87) | 0.35 | 0.00% |
| Depression | Jumping from heights | Asia | 2 | 2.12  (1.94-2.33) | <0.001 | 0.00% |
|  |  | Europe | 1 | 1.73  (1.01-2.95) | 0.04 | 0.00% |
|  | Fire | Asia | 1 | 0.49  (0.40-0.60) | <0.001 | 0.00% |
|  |  | Europe | 1 | 0.87  (0.45-1.65) | 0.66 | 0.00% |
|  | Firearms | North America | 4 | 1.00  (0.16-6.05) | 0.99 | 99.93% |
|  |  | Europe | 1 | 1.00  (0.05-22.18) | 1.00 | 0.00% |
|  | Drowning | Asia | 1 | 0.94  (0.79-1.11) | 0.45 | 0.00% |
|  |  | Europe | 1 | 0.38  (0.02-6.34) | 0.50 | 0.00% |
|  | Gas poisoning | North America | 1 | 0.60  (0.53-0.68) | <0.001 | 0.00% |
|  |  | Asia | 1 | 1.11  (0.80-1.54) | 0.52 | 0.00% |
|  | Drug overdose | All studies conducted in Asia | | | | |
|  | Other poisoning | North America | 1 | 2.30  (2.22-2.37) | <0.001 | 0.00% |
|  |  | Asia | 1 | 0.88  (0.74-1.05) | 0.16 | 0.00% |
|  |  | Europe | 1 | 3.67  (0.12-113.73) | 0.46 | 0.00% |
|  | Hanging | North America | 3 | 0.93  (0.56-1.53) | 0.84 | 95.48% |
|  |  | Asia | 2 | 1.03  (0.78-1.36) | 0.79 | 97.30% |
|  |  | Europe | 1 | 1.00  (0.08-12.56) | 1.00 | 0.00% |

**Supplementary Figure 1**: Funnel plot showing standard error versus effect sizes for schizophrenia and drowning as method of suicide

**Supplementary Figure 2**: Funnel plot showing standard error versus effect sizes for schizophrenia and fire as method of suicide

**Supplementary Figure 3**: Funnel plot showing standard error versus effect sizes for schizophrenia and gas poisoning as method of suicide

**Supplementary Figure 4**: Funnel plot showing standard error versus effect sizes for schizophrenia and hanging as method of suicide

**Supplementary Figure 5**: Funnel plot showing standard error versus effect sizes for schizophrenia and jumping from heights as method of suicide

**Supplementary Figure 6**: Funnel plot showing standard error versus effect sizes for schizophrenia and non-gaseous poisoning as method of suicide

**Supplementary Figure 7**: Funnel plot showing standard error versus effect sizes for schizophrenia and firearms as method of suicide

**Supplementary Figure 8**: Funnel plot showing standard error versus effect sizes for schizophrenia and cutting or the use of sharp objects as method of suicide

**Supplementary Figure 9**: Funnel plot showing standard error versus effect sizes for schizophrenia and non-violent methods of suicide

**Supplementary Figure 10**: Funnel plot showing standard error versus effect sizes for schizophrenia and violent methods of suicide

**Supplementary Figure 11**: Funnel plot showing standard error versus effect sizes for bi-polar and hanging method of suicide

**Supplementary Figure 12**: Funnel plot showing standard error versus effect sizes for bi-polar and non-gaseous poisoning method of suicide

**Supplementary Figure 13**: Funnel plot showing standard error versus effect sizes for bi-polar and non-violent methods of suicide

**Supplementary Figure 14**: Funnel plot showing standard error versus effect sizes for bi-polar and violent methods of suicide

**Supplementary Figure 15**: Funnel plot showing standard error versus effect sizes for depression and hanging method of suicide

**Supplementary Figure 16**: Funnel plot showing standard error versus effect sizes for depression and jumping from heights method of suicide

**Supplementary Figure 17**: Funnel plot showing standard error versus effect sizes for depression and non-gaseous poisoning method of suicide

**Supplementary Figure 18**: Funnel plot showing standard error versus effect sizes for depression and use of firearms as a method of suicide

**Supplementary Figure 19**: Funnel plot showing standard error versus effect sizes for depression and non-violent methods of suicide

**Supplementary Figure 20**: Funnel plot showing standard error versus effect sizes for depression and violent methods of suicide
